# Oxidative Regulation of Vascular Ca_v_1.2 Channels Triggers Vascular Dysfunction in Hypertension-Related Disorders

**DOI:** 10.3390/antiox11122432

**Published:** 2022-12-09

**Authors:** Xiang-Qun Hu, Lubo Zhang

**Affiliations:** Lawrence D. Longo MD Center for Perinatal Biology, Department of Basic Sciences, Loma Linda University School of Medicine, Loma Linda, CA 92350, USA

**Keywords:** hypertension, gestational diabetes, preeclampsia, reactive oxygen species, Ca_v_1.2, myogenic tone

## Abstract

Blood pressure is determined by cardiac output and peripheral vascular resistance. The L-type voltage-gated Ca^2+^ (Ca_v_1.2) channel in small arteries and arterioles plays an essential role in regulating Ca^2+^ influx, vascular resistance, and blood pressure. Hypertension and preeclampsia are characterized by high blood pressure. In addition, diabetes has a high prevalence of hypertension. The etiology of these disorders remains elusive, involving the complex interplay of environmental and genetic factors. Common to these disorders are oxidative stress and vascular dysfunction. Reactive oxygen species (ROS) derived from NADPH oxidases (NOXs) and mitochondria are primary sources of vascular oxidative stress, whereas dysfunction of the Ca_v_1.2 channel confers increased vascular resistance in hypertension. This review will discuss the importance of ROS derived from NOXs and mitochondria in regulating vascular Ca_v_1.2 and potential roles of ROS-mediated Ca_v_1.2 dysfunction in aberrant vascular function in hypertension, diabetes, and preeclampsia.

## 1. Introduction

Free intracellular Ca^2+^ functions as an important second messenger to regulate various physiological processes including muscle contractility, gene expression, hormone secretion, and neuronal transmission [1,2]. The Ca^2+^ signal transduction is initiated by increasing intracellular Ca^2+^ concentration ([Ca^2+^]_i_) due to Ca^2+^ entry through ion channels on the cell membrane and Ca^2+^ release via Ca^2+^-release channels on the endo/sarcoplasmic reticulum. Ca^2+^ influx in vascular smooth muscle cells is primarily mediated by L-type voltage-gated Ca^2+^ (Ca_v_1.2) channels encoded by the *CACNA1C* gene [3]. Vascular tone in small arteries and arterioles, a major determinant of blood pressure and blood flow, is governed by the contractile state of vascular smooth muscle cells which is determined by dynamic changes in intracellular [Ca^2+^]_i_ [4]. An increase in [Ca^2+^]_i_ instigates vascular smooth muscle cell contraction, whereas a decrease in [Ca^2+^]_i_ promotes relaxation.

Elevated blood pressure is a prime characteristic of hypertension and preeclampsia [5,6,7]. Preeclampsia is a multisystem disorder characterized by the onset of hypertension after 20 weeks of gestation. In addition, there is a strong epidemiologic association of diabetes and hypertension, and hypertension is common among diabetic patients [8,9,10]. Prevalence of hypertension in adults is ~30% worldwide in 2010 and in United States between 2011–2012 [11,12,13]. Remarkably, prevalence of hypertension in adults with diabetes is 66–76% in United States [10]. Preeclampsia affects ~5% of pregnancies globally [14]. Hereafter, these three disorders are collectively referred as hypertension-related disorders in this review. Hypertension is categorized into essential (primary) and secondary hypertension. Essential hypertension accounts for 95% human cases and has no known cause, whereas the remaining are secondary hypertension which occurs due to known diseases or conditions including renovascular disease, aldosteronism, Cushing’s syndrome, thyroid dysfunction, and among others [15,16]. Diabetes is commonly classified into two types. Type 1 diabetes results from insulin deficiency due to autoimmune destruction of β-cells in the pancreas, whereas type 2 diabetes originates from insulin resistance owing to reduced insulin sensitivity and accounts for >90% of all cases [17,18]. Hypertension is the leading cause of cardiovascular disease worldwide, including stroke, coronary artery disease, heart failure, arrhythmia, and peripheral vascular disease [19].

High blood pressure arises from vascular dysfunction [20]. Oxidative stress is a hallmark of hypertension, preeclampsia, and diabetes [21,22,23]. Excessive reactive oxygen species (ROS) play an important role in vascular dysfunction and contribute to the pathogenesis of hypertension-related disorders [24,25,26]. Given the pivotal role of Ca_v_1.2 in regulating vascular tone, we discuss in this review the oxidative modification leading to alternations of vascular Ca_v_1.2 function/expression and enhanced myogenic tone in hypertension-related disorders.

## 2. Ca_v_1.2 in Vascular Smooth Muscle

### 2.1. Overview of Ca_v_1.2

**Figure 1:**
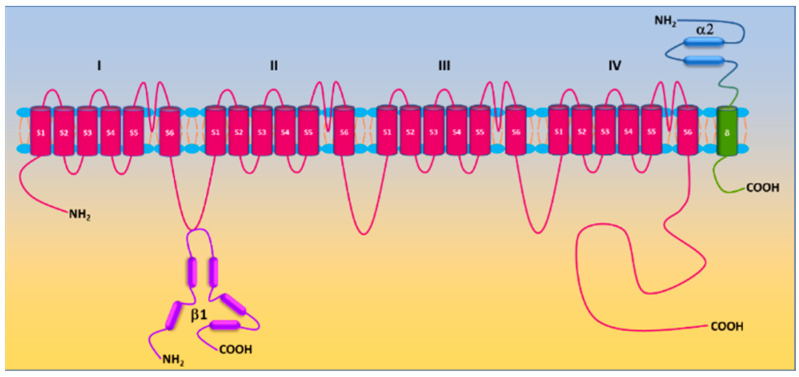
Topology of Ca_v_1.2. Ca_v_1.2 is formed by the pore-forming transmembrane α1c subunit, the intracellular β-subunit, the extracellular α2 subunit linked to the transmembrane δ1 subunit in smooth muscle cells.

L-type Ca^2+^ (Ca_v_) channels are heteromultimeric complexes comprising pore-forming α1c and auxiliary β, α2δ, and γ subunits [27]. The α1c subunit possesses four repeat domains (I–IV) linked by intracellular loops and intracellular NH_2_-/COOH-termini with each domain containing six transmembrane segments (S1–S6) (Figure 1). The ion-conducting pore is formed by S5 and S6 and the loop between them, whereas the voltage sensor is located in the S4 segments [28]. The intracellular COOH terminus, along with intracellular loops, plays important roles in Ca^2+^-dependent inactivation, channel trafficking, phosphorylation and oxidation [28,29,30]. Ca_v_ is activated by membrane depolarization and its activation allows Ca^2+^ influx through the channel pore. The expression of the α1c subunit itself can form functional channels to conduct Ca^2+^ ions, whereas the incorporation of auxiliary subunits promotes membrane α1c expression and alters biophysical properties of the channel [28,31]. Ca_v_ channels are sensitive to blockades by dihydropyridines (i.e., nifedipine), phenylalkylamines (i.e., verapamil) and benzothiazepines (i.e., diltiazem) [32]. There are four types of Ca_v_ channels (Ca_v_1.1–1.4). Ca_v_1.2 is predominantly expressed in the heart and in vascular smooth muscle [33,34]. Ca^2+^ influx through the channel is a primary trigger of vasoconstriction and also participates in transcriptional regulation. The auxiliary subunits β2, β3, and α2δ1 are expressed in vascular smooth muscle [35,36,37,38]. β subunits, lacking membrane-spanning segments, are located intracellularly and interact with the α interaction domain (AID) on the I-II linker of the α1c subunit [39]. The α2δ1 subunit is a single gene product bound together by disulfide bonds. Whereas the α2 subunit is extracellular, the δ subunit contains a single transmembrane segment [40]. The diversity of Ca_v_1.2 is also conferred by alternative splicing. Smooth muscle contains Ca_v_1.2 exons 1, 8, 9 *, 31/32, and 33 [41,42]. Due to the scope of this review, only a brief description of Ca_v_1.2 is presented here. The reader is referred to the literature for detailed information on Ca_v_1.2 [43,44,45].

### 2.2. Regulation of Ca_v_1.2

#### 2.2.1. Regulation by Auxiliary Subunits

The molecular composition of Ca_v_1.2 in vascular smooth muscle cells includes the pore-forming α1c subunit and auxiliary β2/3 and α2/δ1 subunits. The β3 subunit appears to be the principal β isoform in vascular smooth muscle cells [35]. Genetic deletion of the β3 subunit resulted in reduced α1c expression in mouse aorta and is associated with a reduction in Ca^2+^ channel current and a slower inactivation rate [35]. This genetic manipulation also attenuates angiotensin II-induced upregulation of Ca_v_1.2 channels in mouse mesenteric arteries and the development of hypertension [38]. Similarly, knocking-down α2/δ1 with short hairpin RNA in cerebral artery reduced plasma membrane α1c expression [36]. Moreover, Ca_v_1.2 currents were inhibited by pregabalin, an α2/δ1/2 ligand, and by an α2/δ-1 antibody in cerebral vascular smooth muscle cells, leading to vasodilation [36].

#### 2.2.2. Regulation by Protein Kinases

Protein phosphorylation, a covalent addition of the phosphate group to the side chain of serine, threonine, and tyrosine residues by protein kinases, is a common posttranslational modification to fine tune activities of receptors, ion channels and enzymes. Unsurprisingly, Ca_v_1.2 is a target of protein kinases, and its activity is subject to the regulation by protein phosphorylation. Both α1c and β2 subunits are phosphorylated by protein kinases A (PKA), C (PKC), and G (PKG) [29,46,47,48,49]. A variety of putative serine/threonine phosphorylation sites have been identified, yet their role in regulating Ca_v_1.2 remains unsettled [50]. In vascular smooth muscle cells, the regulation of Ca_v_1.2 by PKA is controversial. PKA is found to either inhibit or enhance Ca_v_1.2 activity [51,52,53,54,55,56,57]. The stimulatory effect of PKA on Ca_v_1.2 in vascular smooth muscle cells depends on anchoring adenyl cyclase 5 and PKA by A-kinase anchoring protein 150 (AKAP150) to the proximity of Ca_v_1.2 [56,58]. Phosphorylation of Ser 1928 in the COOH-terminus of the Ca_v_1.2 α1c subunit is required for PKA-stimulated channel activity in vascular smooth muscle cells [56]. Activation of PKG exhibits inhibitory effects on vascular Ca_v_1.2 [52,57,59,60]. PKG mediates nitric oxide-induced inhibition of Ca_v_1.2 [61,62]. Activation of PKC by phorbol esters and by Gq-coupled receptors also potentiates vascular Ca_v_1.2 activity [63,64,65,66,67,68,69,70]. Basal Ca_v_1.2 activity is evidently under the tonic control of PKC as PKC inhibition/PKCα depletion enhances Ca_v_1.2 activity in vascular smooth muscle cells [67]. Activation of PKG by nitric oxide (NO) suppresses Ca_v_1.2 activity [49]. Similar to PKA, PKC is anchored by AKAP150 to adjacent Ca_v_1.2 to alter channel activity [71]. Protein tyrosine kinase c-Src promotes tyrosine phosphorylation of the α1c subunit and enhances Ca_v_1.2 activity, which is believed to participate in regulating smooth muscle contractility [72]. c-Src via its SH_2_ and SH_3_ domains binds to the COOH-terminus of the α1c subunit [70]. Y2122 in the COOH-terminus of the α1c subunit appears to be the major phosphorylation site of c-Src [70,73]. In vascular smooth muscle cells, c-Src enhances Ca_v_1.2 activity [64,74,75,76]. Phosphoinositide 3-kinases (PI3Ks) are found to increase Ca_v_1.2 activity in vascular smooth muscle cells [77,78]. PI3Kγ potentiates Ca_v_1.2 activity by facilitating plasma membrane translocation of α1c subunits and this effect is mediated by AKT/PKB-induced β2 subunit phosphorylation [79,80,81,82]. Integrins also participate in the mechanotransduction process of pressure-induced myogenic tone [83]. Integrin receptor activation is found to increase Ca_v_1.2 activity via c-Src and PKA [84,85,86].

#### 2.2.3. Regulation by Small GTPases

The Ras superfamily of small GTPases (also known as small G-proteins) are cellular switches that regulate a variety of biological processes in living cells. They have been implicated in regulating Ca^2+^ homeostasis and Ca_v_1.2 is recognized as an important effector of the RGK subfamily (Rem, Rem2, Rad, and Gem/Kir) [87,88]. Unlike vascular Ca_v_1.2, direct phosphorylation of the cardiac Ca_v_1.2 α1c subunit does not contribute to PKA-stimulated channel activity as mutating all PKA consensus sites in the α1c subunit fails to block the increase in Ca_v_1.2 activity in response to β-adrenergic stimulation [89,90]. Cardiac Ca_v_1.2 is under tonic inhibition of Rad due to its association to both α1c and/or β subunits [91,92]. β-adrenergic stimulation induces RAD phosphorylation, promotes the release of RAD from Ca_v_1.2, and increase channel activity [90]. Similarly, Ca_v_1.2 activity is suppressed by other members of the RGK subfamily [93,94,95]. It remains to be determined whether vascular Ca_v_1.2 is modulated by RGK members. Small GTPases also regulate surface expression of Ca_v_1.2. Rab25, a member of the Rab subfamily, regulates intracellular vesicle trafficking and promotes surface expression of Ca_v_1.2 in vascular smooth muscle cells [96].

### 2.3. Ca_v_1.2 and Myogenic Tone

Vascular smooth muscle cells in resistant arteries and arterioles possess intrinsic ability to contract in response to an increase in intraluminal pressure and to relax upon a decrease in intraluminal pressure [97]. This phenomenon is defined as myogenic response and the steady-state of vascular smooth muscle cell contractility in these vessels is termed as myogenic tone. Myogenic tone sets the basal vascular tone and distribution of blood flow to and within tissues/organs. In principle, peripheral vascular resistance can be described by the Poiseuille equation: R = 8Lη/πr^4^, where R is the resistance, L is length of the vessel, η is viscosity of blood, and r is the radius of the vessel. According to the Poiseuille’s law, peripheral vascular resistance is inversely proportional to the radius to the fourth power. For example, decreases in vessel caliber by 10% and 20% will theoretically lead to increases in vascular resistance by ~50% and ~145%, respectively. Thus, the radius of a given small artery/arteriole has a great impact on peripheral vascular resistance and blood flow.

**Figure 2:**
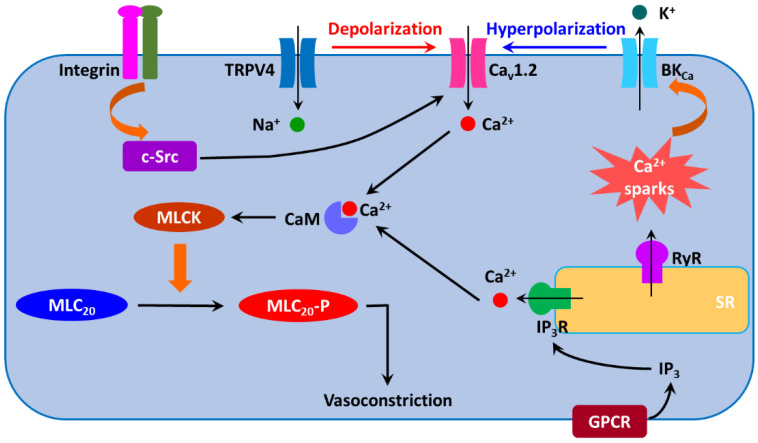
Proposed mechanisms for regulating myogenic tone in small arteries and arterioles. Detailed information is discussed in the text. GPCR, G protein-coupled receptor, TRPV4, Transient receptor potential vanilloid-type 4; BK_Ca_, large-conductance Ca^2+^-activated K^+^ channel; SR, sarcoplasmic reticulum; IP_3_, inositol triphosphate; RyR, ryanodine receptor; CaM, calmodulin; MLCK, myosin light-chain kinase; MLC_20_, 20 kD myosin light-chain.

Vascular smooth muscle cells in resistance arteries/arterioles become depolarized in response to increased intraluminal pressure [98,99,100]. Various mechanisms have been proposed to regulate myogenic tone (Figure 2). It is widely believed that pressure-induced membrane depolarization in vascular smooth muscle cells is instigated by mechanosensitive or stretch-activated cation channels including transient receptor potential (TRP) channels and epithelial Na^+^ channels (ENaCs) [101,102,103,104,105,106,107]. The membrane depolarization leads to increased [Ca^2+^]_i_ and vasoconstriction [99,108]. The increases in both [Ca^2+^]_i_ and/or myogenic tone are blocked by the removal of extracellular Ca^2+^ or by Ca_v_1.2 blockers [99,109,110,111,112,113,114,115]. These findings suggest that altered intraluminal pressure initiates myogenic tone via membrane depolarization and subsequent opening of Ca_v_1.2. This notion is corroborated by findings from the genetic deletion of Ca_v_1.2 α1c subunit in smooth muscle [116]. Such a manipulation abolishes myogenic reactivity in murine tibialis arteries [116]. However, Ca^2+^ influx mediated by T-type voltage-gated Ca^2+^ channels (Ca_v_3.x) may also contribute to myogenic tone development [106,117]. Integrins, linking the extracellular matrix and cellular cytoskeleton, could also function as mechanotransducer to regulate myogenic tone via c-Src-mediated phosphorylation of Ca_v_1.2 [83,85,118]. Moreover, some G protein-coupled receptors are mechanosensitive and produce inositol triphosphate (IP_3_) upon stretch to induce Ca^2+^ release via activating IP_3_R on the sarcoplasmic reticulum (SR) membrane [119,120]. Over myogenic tone, extrinsic factors such as vasodilators and vasoconstrictors can induce vasodilation and further vasoconstriction, respectively. It should be noted that the large-conductance Ca^2+^-activated K^+^ channel is also activated by the increase in intralumenal pressure, which functions as a negative feedback mechanism to limit the magnitude of intralumenal pressure-induced vasoconstriction under physiological conditions [121]. For more detailed information on the regulation of myogenic tone by ion channels, readers are referred to recent reviews on this topic [106,122,123]. As aforementioned, myogenic response is the intrinsic property of vascular smooth muscle cells to respond to changes in intraluminal vascular pressure. In general, myogenic tone development is independent of endothelium [124,125,126]. However, myogenic tone can be modified by the endothelium. For example, activation of the endothelial intermediate-conductance Ca^2+^-activated K^+^ channel is found to reduce myogenic tone [127,128]. Moreover, myogenic tone in mouse mesenteric and uterine arteries from pregnant mice is enhanced by the loss of endothelium-derived NO [129].

## 3. Roles of Ca_v_1.2 in the Pathogenesis of Hypertension-Related Disorders

### 3.1. Aberrant Vascular Tone in Hypertension-Related Disorders

Hypertension is associated with increased peripheral vascular resistance. As myogenic tone is the fundamental element of vascular tone, it is reasonable to speculate that myogenic tone is altered in hypertension-related disorders. In patients with hypertension, myogenic tone is increased in coronary arterioles [110]. Increased myogenic tone is also noted in resistance arteries from the adipose tissue of paravertebral muscles of hypertensive patients [130]. Commonly used rat models of experimental hypertension include the spontaneously hypertensive rat (SHR), stroke-prone spontaneously hypertensive rat (SHRSP), Milan hypertensive strain (MHS), vasopressin-deficient (Di/H) rats, two-kidney, one-clip (2K1C rats), and among others. Compared to the Wistar-Kyoto rat (WKY), myogenic tone of afferent arterioles and arcuate arteries from SHR kidneys is increased [131,132]. Mesenteric arteries of SHR and MSH also exhibits higher myogenic tone than those of WKY and Milan normotensive strain (MNS), respectively [133,134]. Elevated myogenic tone is observed in cerebral arteries/basilar arteries of SHR/SHRSP/Di/H compared to WKY and Di normotensive (Di/N) rats, respectively [135,136,137,138]. Similarly, there is an increase in the myogenic tone of cremaster arterioles of SHR [139,140]. Furthermore, chronic infusion of angiotensin II in C57BL/6 mice also causes increased myogenic tone in middle cerebral arteries [141]. In contrast, mice with smooth muscle cell-specific deficiency of the mineralocorticoid receptor reduces myogenic tone and lowers blood pressure [142,143].

Myogenic tone is increased in skeletal muscle resistance arteries of diabetic patients [144]. Various animal models such as type I diabetic rodents induced by streptozotocin (STZ), obese/type II diabetic rodents induced by high-fat diet (HFD), type II diabetic HFD/STZ rodents, type II diabetic Goto-Kakizaki (GK) rats, and type II diabetic C57BL/KsJ-db/db mouse have been developed in diabetes research [145,146]. Systemic vascular resistance (also known as total vascular resistance) and arterial blood pressure are increased in C57BL/KsJ-db/db mice, HFD rats, and GK rats [147,148,149,150,151,152,153,154,155]. Mesenteric arteries of HFD mice, HFD/STZ mice, and diabetic (db/db) mice displayed higher myogenic tone compared to their counterparts [58,144,155,156]. Similarly, the cerebral arteries of STZ-Sprague Dawley rats, Bio-Breeding Zucker diabetic (BBZDR/Wor) rats, HFD mice, and HFD/STZ mice also exhibit increased myogenic tone [144,157,158]. Moreover, higher myogenic tone is detected in gracilis arterioles of STZ Wistar rats and db/db mice [148,159]. Furthermore, myogenic tone of cerebral arteries and ophthalmic arteries from C57BL/6J WT mice and Sprague Dawley rats increases in response to acute changes of glucose levels from low (10 mM) to high concentrations (20–25 mM, to mimic hyperglycemia) [58,160,161].

A study by Kublickiene and colleagues find that preeclampsia does not alter myogenic tone of myometrial arteries [162]. However, the measurement is made in only one pressure point. Nevertheless, the authors reveal that flow-mediated dilatation is lost in pressurized myometrial arteries from women with preeclampsia [162]. A frequently used rodent model of preeclampsia is induced by chronic reductions in uteroplacental perfusion (RUPP) in rats/mice by clipping/ligating the aorta below the renal arteries or the arterial and venous branches of the uterine vascular arcade [163,164]. Myogenic tone is increased in both uterine arteries and mesenteric arteries in this animal model [165,166,167]. Intriguingly, exposure to small extracellular vesicles purified from the plasma of pregnant women with preeclampsia elevates myogenic tone in the mesenteric arteries of C57Bl/6J mice [168], suggesting that circulating bioactive factor(s) could contribute to the increased peripheral vascular tone in preeclampsia. Hypoxia plays a key role in the pathogenesis of preeclampsia [169,170,171]. High-altitude hypoxia increases maternal systolic and diastolic blood pressure at term in human pregnancy [172] and is associated with increased incidence of preeclampsia [173,174,175]. Pregnant sheep exposed to normobaric hypoxia at low altitude and hypobaric hypoxia at high altitude developed preeclampsia-like symptoms including elevated maternal systemic blood pressure and increased uterine vascular resistance as well as various biochemical changes in the circulation and uteroplacental tissues [176,177,178]. Myogenic tone of resistance uterine arteries is increased in pregnant sheep acclimatized to high-altitude hypoxia [179]. This effect is replicated by ex vivo hypoxia treatment of resistance uterine arteries of low-altitude pregnant sheep [180].

### 3.2. Dysfunction of Vascular Ca_v_1.2 in Hypertension-Related Disorders

As aforementioned, activation of Ca_v_1.2 in vascular smooth muscle cells is essential for the development of myogenic tone [99,100,109,110,113,114,116]. The enhanced myogenic tone in peripheral resistance arteries and arterioles in hypertension-related disorders suggests potential dysfunction of vascular Ca_v_1.2. Indeed, this notion is substantiated by lines of evidence from functional studies. First, the increased myogenic tone in resistance arteries/arterioles of hypertensive and diabetic animals was normalized by the Ca_v_1.2 blocker nifedipine [58,132,181]. Whereas specific deletion of the mineralocorticoid receptor reduces KCl- and Ca_v_1.2 agonist Bay K 8644-induced vasoconstriction of mesenteric arteries [142,182], Bay K 8644-induced contraction and increase in [Ca^2+^]_i_ in 2K1C rat aorta is greater than in the control 2K rats [183]. Second, Bay K 8644 triggers greater contraction of inferior epigastric arteries of women with preeclampsia than that of normotensive subjects [184]. Similarly, Bay K 8644- or the membrane-depolarizing agent KCl-induced contraction of cerebral arteries, mesenteric arteries, renal arteries, femoral arteries is greater in SHR, diabetic STZ or GK rats, RUPP rats or pregnant rats treated with nitric oxide synthase inhibitor L-N^ω^-nitro arginine methyl ester (L-NAME) than their control counterparts [153,185,186,187,188,189,190,191]. Third, Bay K 8644 or KCl produces a larger increase in [Ca^2+^]_i_ and contraction in renal arteries in RUPP rats and pregnant rats treated with L-NAME [186,188].

Animal models of hypertension-related disorders have provided mechanistic insights into the understanding of the Ca_v_1.2 dysfunction in these disorders. Both aberrant expression of and dysregulation of Ca_v_1.2 contribute to vascular Ca_v_1.2 dysfunction. Various studies reveal increased protein expression of α1c [37,38,187,192,193], α2δ1 [37,192], and β3 [37,38] subunits in mesenteric, femoral, and cerebral arteries of SHR and angiotensin II-infused C57BL/6 mice. Consistently, increased expression of Ca_v_1.2 is associated with enhanced channel activity in vascular smooth muscle cells [67,185,193,194,195,196,197]. The expression and activity of Ca_v_1.2 are reduced in mesenteric arteries of aged mice lacking mineralocorticoid receptors in smooth muscle cells [143]. Dexamethasone administration increases the expression of the cardiac Ca_v_1.2 α1c subunit in rats [198]. As expected, Ca_v_1.2 activity in A7r5 cells is increased following chronic dexamethasone exposure [199]. Similarly, hyperthyroidism also boosts the expression of cardiac Ca_v_1.2 [200]. Galectins (Gals) are a family of carbohydrate-binding lectins. Gal-1, through binding preferentially to Ca_v_1.2 I–II loop without exon 9*, reduces surface expression of Ca_v_1.2 in vascular smooth muscle cells [201]. Gal-1 is later found to compete with the β subunit for binding to the α1c subunit leading to polyubiquitination and degradation of the α1c subunit and consequent reduction of Ca_v_1.2 activity [202]. Notably, protein levels of Gal-1 and Ca_v_1.2 are downregulated and upregulated, respectively, in arteries of SHR and hypertensive patients [202]. In hypertensive human pulmonary arteries, the downregulation of Gal-1 is mediated by hypoxia-inducible factor 1α [202]. The knockdown of Gal-1 with siRNA increases Ca_v_1.2 activity and promotes vasoconstriction [201], whereas the deletion of the Gal-1 encoding gene *LGALS1* increases blood pressure [202].

An increase in protein expression of the α1c subunits is also observed in cerebral arteries of STZ rats, which is associated with increased Ca_v_1.2 activity and contraction to KCl [203]. Ca_v_1.2 also displays increased activity in vascular smooth muscle cells of cerebral and mesenteric arteries from STZ, GK, and HFD rats and db/db mice [153,204,205,206]. Human arteries from diabetic patients have higher Ca_v_1.2 activity due to increased phosphorylation of α1C at Ser1928 by PKA [56]. Similar findings were observed in cerebral arteries of HFD mice [56]. These changes are simulated by in vitro acute exposure of rodent and human arteries to a high concentration of glucose [56,205]. A series of studies by Navedo’s group reveal that the Gs-coupled purinergic receptor P2Y11, adenylyl cyclase 5 (AC5), PKA, AKAP150, and Ca_v_1.2 form a signaling microdomain in vascular smooth muscle cells and these components are localized in nanometer proximity within the microdomain [56,58,161]. Apparently, elevated glucose stimulates Ca_v_1.2 activity by promoting autocrine release of nucleotides, which sequentially activates P2Y11, AC5, and PKA leading to increased phosphorylation of the α1C subunit at Ser1928 [56,58,161].

Gal-1 is highly expressed in the placenta [207]. Circulating Gal-1 level also increases during gestation [208]. Given the critical role of Gal-1 in regulating Ca_v_1.2 surface expression/activity discussed above, it is reasonable to speculate that the elevated Gal-1 in pregnancy may contribute to reduced uterine arterial myogenic tone by suppressing Ca_v_1.2 surface expression/activity [209,210]. Intriguingly, both circulating and placental Gal-1 is downregulated in early onset preeclampsia and pregnant women with fetal growth restriction [211,212]. The downregulation of Gal-1 probably plays a role in the increased myogenic tone in uterine and other vascular beds in preeclampsia due to upregulating Ca_v_1.2 surface expression/activity [165,166,167,179].

## 4. Roles of Reactive Oxygen Species (ROS) in the Pathogenesis of Hypertensive Disorders

### 4.1. Overview of ROS

Reactive oxygen species (ROS) are oxygen-containing molecules naturally produced in cellular metabolism. ROS comprise free radicals such as superoxide anion (O_2_^•−^) and hydroxyl radical (^•^OH) and nonradical molecule hydrogen peroxide (H_2_O_2_). O_2_^•−^ is formed from the one-electron reduction of molecular oxygen (O_2_). It is a precursor to a cascade of other ROS. Its dismutation, either occurring spontaneously or being catalyzed by superoxide dismutases (SODs), produces H_2_O_2_. Through the Fenton reaction, H_2_O_2_ can be reduced to ^•^OH. Moreover, the reaction between O_2_^•−^ and nitric oxide (NO) results in the formation of peroxynitrite (ONOO^−^).

**Figure 3:**
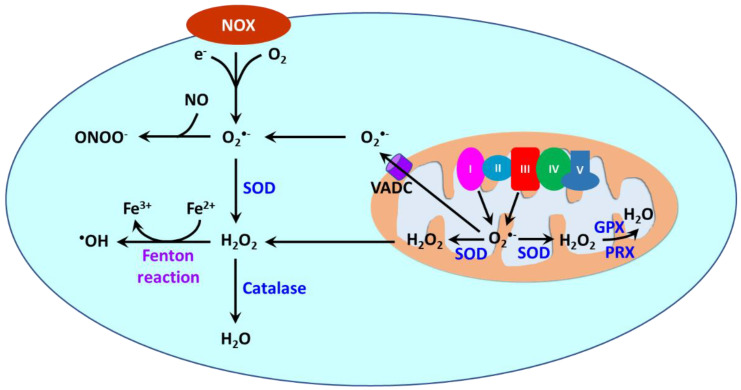
ROS generation by NOXs and mitochondria and detoxification. In vascular smooth muscle cells, ROS is primarily produced by NOXs and mitochondria. NOXs catalyze the production of O_2_^•−^ by transferring one electron to O_2_ from NADPH. In mitochondria, O_2_^•−^ is also produced from leaked electrons by Complexes I and III in the electronic transfer chain (ETC) during the electronic transfer. O_2_^•−^ is dismutated to H_2_O_2_ by superoxide dismutase (SOD). H_2_O_2_ is subsequently decomposed to H_2_O by catalase, glutathione peroxidase (GPX) and peroxiredoxin (PRX). O_2_^•−^ can be released into the cytosol from mitochondria via the voltage-dependent anion channel (VADC) on the mitochondrial outer membrane, whereas H_2_O_2_ in mitochondria can be transported to the cytosol via diffusion or via aquaporins. O_2_^•−^ reacts with nitro oxide (NO) to produce peroxynitrite (ONOO^−^), whereas H_2_O_2_ reacts with Fe^2+^ via the Fenton reaction to yield ^•^OH.

ROS are produced in different cellular compartments including mitochondria, endoplasmic reticulum (ER), lysosomes, peroxisomes, and plasma membrane [23,213,214]. Nicotinamide adenine dinucleotide phosphate oxidases (NOXs) and mitochondria are the major sources of ROS [215] (Figure 3). NOXs are a family of transmembrane proteins that catalyze the transfer of electron donated by NADPH to molecular oxygen to form O_2_^•−^ [216]. NOXs comprise seven isoforms (NOX1-5 and dual oxidases (DUOX) 1 and 2). NOX1, NOX2, NOX4, and NOX5 are primary isoforms in vasculature [217]. During oxidative phosphorylation, ATP synthesis is coupled to the movement of electrons derived from NADH and FADH_2_ through Complexes I to IV in the mitochondrial electron transport chain (ETC). Electrons are received by O_2_ at Complex IV to produce H_2_O. Mitochondria consumes ~90% cellular O_2_ [218]. It is estimated that ~1–2% of O_2_ consumed by mitochondria reacted with leaked electrons, leading to the formation of O_2_^•−^ [219]. Complexes I and III are the major sites in ETC to generate O_2_^•−^ [220].

ROS can be beneficial and detrimental. They are highly reactive. O_2_^•−^ and ^•^OH are short-lived and exert their actions in their immediate vicinity. In contrast, H_2_O_2_ is stable and can travel a long distance in cells and pass through the membrane. While O_2_^•−^ and ^•^OH nonspecifically act on macromolecules such as proteins, DNA, and lipids, H_2_O_2_ can trigger reversible oxidation of cysteine residues in specific proteins including enzymes, protein kinases, transcription factors, and ion channels, leading to altered protein functions [221,222]. Thus, H_2_O_2_ is considered as an intracellular signaling molecule at physiological concentrations [221,222]. However, H_2_O_2_ causes irreversible damage to macromolecules and impairs their functions at supraphysiological concentrations.

Cellular ROSs are tightly regulated under physiological conditions. Physiological ROS concentrations are maintained by various antioxidant mechanisms. O_2_^•−^ is converted into H_2_O_2_ by Cu, Zn-SOD (SOD1) in the cytosol/mitochondrial intermembrane space and Mn-SOD (SOD2) in the mitochondrial matrix. H_2_O_2_ is subsequently decomposed to H_2_O by catalase, glutathione peroxidases (GPXs) and peroxiredoxins (PRXs). ROS can also be scavenged by nonenzymatic antioxidants such as glutathione (GSH), vitamins C/E, uric acid, and melatonin [223]. Oxidative stress occurs when the ROS homeostasis is disrupted due to ROS overproduction, or antioxidant deficiency, or both. Thus, oxidative stress represents a state of an imbalance between ROS production and antioxidant defenses.

### 4.2. Oxidative Stress as a Hallmark in Hypertension-Related Disorders

Hypertension, diabetes, and preeclampsia exhibit a state of oxidative stress [21,22,23]. A variety of studies have demonstrated that levels of biomarkers of oxidative stress such as O_2_^•−^/H_2_O_2_, malondialdehyde, 8-isoprostaglandin F2α(8-iso-PGF2α), oxidized low-density lipoprotein (ox-LDL), and protein carbonyl are increased, whereas levels of antioxidants GSH (or GSH/GSSG ratio), vitamin C/E, and levels/activities of SOD, catalase, and GRX are decreased in the circulation of hypertensive [224,225,226,227,228,229,230,231], diabetic [232,233,234,235,236,237,238,239], and preeclamptic [240,241,242,243,244,245,246,247,248] patients. In addition, urinary 8-iso-PGF2α excretion is also increased in these disorders [231,249,250]. Systemic oxidative stress has also been detected in animal models of hypertension [251,252,253,254,255,256,257,258], diabetes [259,260,261,262], and preeclampsia [263,264,265].

Notably, hypertension-related disorders also undergo vascular oxidative stress. Both O_2_^•−^ and H_2_O_2_ are increased in vascular cells in experimental models of hypertension including SHR/SHRSP rats, DOCA rats, angiotensin II-induced mice, and transgenic mice overexpressing smooth muscle cell-specific NOX1 [266,267,268,269,270,271,272,273,274,275]. In addition, SHR and angiotensin II-infused mice display elevated vascular lipid peroxidation, 8-hydroxydeoxyguanosine, and nitrotyrosine [252,276]. Chronic administration of aldosterone increases O_2_^•−^ production in mouse cerebral arteries and aortas [277,278]. Moreover, specific deletion of the mineralocorticoid receptor suppresses angiotensin II-induced vascular ROS [142,143]. Diabetic patients have elevated vascular O_2_^•−^ [279]. Similarly, vascular O_2_^•−^ and H_2_O_2_ are increased in db/db mice and GK rats [280,281,282]. The increased vascular ROS are simulated by exposing vascular smooth muscle cells and endothelial cells to high glucose [283,284,285,286,287]. O_2_^•−^ overproduction and/or oxidative stress biomarkers nitrotyrosine and 4-hydroxnonenal (4-HNE) are observed in the placenta and vasculature of women with preeclampsia [288,289,290,291,292]. Placental and vascular oxidative stress are also detected in animal models of preeclampsia including catechol-O-methyltransferase deficient (COMT^−/−^) mice, endothelial nitric oxide synthase deficient (eNOS^−/−^) mice, RUPP rats, rodents/sheep experiencing gestational hypoxia, and rodents infused with preeclampsia-related bioactive factors [264,265,293,294,295,296,297,298,299,300,301,302,303]. Exposure to plasma from women with preeclampsia promotes O_2_^•−^ production in uterine arteries and vascular cells [246,304]. Ex vivo hypoxia also induces heightened oxidative stress in uterine arteries [305].

ROS overproduction by NOXs and mitochondria are major sources leading to the heightened oxidative stress in vasculature of human hypertensive patients and experimental animal models of hypertension-related disorders, although suppressed activity and/or expression of SODs, catalase, GPX, TRX, and TRXR also contribute [276,286,288,289,290,293,306,307,308,309].

#### 4.2.1. NOX-Derived ROS in Hypertension-Related Disorders

Several polymorphisms in the gene encoding p22^phox^ in human are associated with hypertension by affecting enzymatic activity [310]. In SHR/SHRSP, the expression/activity of vascular NOXs 1, 2 and 4 are increased [270,272,311,312,313,314,315,316]. DOCA rats have higher vascular expression of NOX subunit p22phox and enzymatic activity [270,317]. NOX activity in cultured vascular smooth cells and in cultured rat mesangial cells is also stimulated by aldosterone, leading to increased ROS generation [318,319]. Chronic administration of aldosterone causes NOX2-depednet increase O_2_^•−^ production in mouse cerebral arteries [277]. Dexamethasone upregulates Nox1 expression in rat aorta via activation of the glucocorticoid receptor [320]. In a mouse model of hyperadrenergic hypertension created by targeted ablation of the chromogranin a (Chga) gene, renal expression of NOX1/2 is increased [321]. Angiotensin II-infused rodents are associated with elevated expression of NOXs 1 and 2 as well as NOX subunits p22^phox^, p47^phox^, and Rac1 in vessels and increased NOX activity [268,274,322,323,324,325,326,327]. Angiotensin II apparently contributes to the upregulation of NOXs as it stimulates gp91^phox^ (NOX2) and p22^phox^ expression in vascular smooth muscle cells from human resistance arteries [328].

In diabetic patients, vascular protein abundance of NOX subunits p22^phox^, p67^phox^, and p47^phox^ and enzymatic activity of NOXs are increased [279]. Moreover, O_2_^•−^ production is reduced by non-selective NOX inhibitor diphenylene iodonium in vessels from diabetic patients [279]. The expression and activity of NOXs 1, 2 and 4 as well as p22^phox^/p47^phox^ are increased in aorta and mesenteric arteries of STZ rodents [287,308,329,330,331,332,333]. Vascular expression/activity of NOXs 1, 2, and 4 as well as p22^phox^ are also elevated in db/db mice [280,334,335]. Chronic high glucose exposure stimulates p22^phox^, p47^phox^ and p67^phox^ expression and NOX activity in cultured endothelial cells [285,286,336,337,338]. Similarly, high glucose also promotes NOXs 1 and 4 as well as p22^phox^ expression in cultured vascular smooth muscle cells [287,308].

In human, the preeclamptic placenta exhibits elevated expression of NOX1, p22^phox^, p47^phox^, and p67^phox^ [308,339]. Preeclampsia is also associated with increased vascular NOX2 expression [304,340]. The expression of NOX2 in ovine uterine arteries is also higher in high-altitude pregnancy [299]. Women with preeclampsia produce excessive angiotensin II type 1-receptor autoantibody (AT1-AA), soluble fms-like tyrosine kinase-1 (sFlt-1), ox-LDL, and tumor necrosis factor-α (TNF-α), leading to high concentrations of them in the circulation [244,341,342,343,344]. In rat models of preeclampsia induced by AT1-AA or sFlt-1 infusion, NOX activity is found to be increased in the placenta, kidney, and aorta [294,345,346,347,348]. sFlt-1 promotes the expression of NOXs 1, 2, and 4 in the placenta and kidney [348]. Circulating activin is also increased in preeclampsia and activin infusion into pregnant mice enhances NOX2 expression in aorta [340,349]. In cultured human trophoblasts, AT1-AA promotes the expression of p22^phox^, p47^phox^ and p67^phox^ and increases O_2_^•−^ generation [350]. In cultured human uterine microvascular endothelial cells and human umbilical vein endothelial cells (HUVECs), preeclamptic plasma/serum increases the expression of NOXs 2 and 4 and NOX activity, which is simulated by sFlt-1, activin, and ox-LDL [292,340,345,351]. The expression of p47^phox^ and p67^phox^ rises and production of O_2_^•−^ increases following AT1-AA treatment in cultured human vascular smooth muscle cells [350]. TNF-α boosts p22^phox^ express and O_2_^•−^ generation in rat aortic smooth muscle cells [352]. Ox-LDL stimulates vascular ROS generation via both upregulating its receptor lectin-like ox-LDL receptor-1 (LOX-1) and stimulating NOX activity [353,354]. Vascular O_2_^•−^ production is increased in RUPP rats in part due to LOX-1 upregulation [296].

#### 4.2.2. Mitochondria-Derived ROS in Hypertension-Related Disorders

Mitochondria become dysfunctional in hypertension-related disorders. SIRT3, a histone deacetylase, is an important regulator of mitochondrial redox state. It interacts with SOD2 in mitochondria and subsequently promotes SOD2 deacetylation, leading to enhanced enzymatic activity [355,356]. Essential hypertension in human is associated with increased mitochondrial oxidative stress in arterioles from mediastinal fat due to reduced SIRT3 and increased SOD2 acetylation [357]. Similar to human essential hypertension, SHR also exhibits overproduction of mitochondrial ROS in vessels [358]. Partial deletion of SOD2 in mitochondria (SOD2^+/−^) increases renal oxidative stress and blood pressure [359]. In angiotensin II-infused mice, the increased blood pressure is associated with increased SIRT3 S-glutathionylation and vascular SOD2 acetylation, reduced SOD2 activity and elevated vascular O_2_^•−^ production [357,360]. Those alterations are diminished by SIRT3 overexpression [357]. Cyclophilin D is a regulatory subunit of the mitochondrial permeability transition pore and participates in regulating mitochondrial function [361]. Evidently, it is an important mediator of angiotensin II-induced hypertension as its depletion diminishes both mitochondrial O_2_^•−^ generation in aorta and hypertension [362]. Mitochondrial O_2_^•−^ generation in DOCA rat mesenteric arteries is also increased [255]. In cultured HUVECs, excess aldosterone suppresses SOD2 expression and increases mitochondrial ROS production [363]. Interestingly, mitochondrial ROS is also regulated by a phenomenon termed ROS-induced ROS release [364]. In cultured human aortic endothelial cells, silence of NOX2 with siRNA reduces angiotensin II-induced mitochondrial O_2_^•−^ generation [365]. Likewise, NOX2 deletion by gp91^phox^ knockout also reduces angiotensin II-induced mitochondrial O_2_^•−^ generation in aorta and hypertension [365].

Elevation of mitochondrial O_2_^•−^ is detected in subcutaneous arterioles in type 2 diabetic patients [366]. Likewise, mitochondrial H_2_O_2_ is increased in primary human saphenous vein endothelial cells from type 2 diabetic subjects [367]. STZ mice/rats, HFD mice, ZDF rats, and GK rats display increased mitochondrial ROS in vascular smooth muscle cells and endothelial cells [331,368,369,370,371,372]. Hyperglycemia is apparently a causative factor leading to heightened mitochondrial ROS in vascular cells. Exposure to high concentrations of glucose stimulates mitochondrial ROS production in a variety of endothelial cells, promoting mitochondrial damage by producing 8-hydroxydeoxyguanosine and nitrotyrosine [373,374,375,376,377,378,379,380,381].

Preeclampsia is associated with reduced mitochondrial content and decreased oxidative phosphorylation in the placenta [382,383]. Preeclamptic placenta also exhibits mitochondrial oxidative stress as evidenced by increased malondialdehyde and 4-HNE-modified proteins in mitochondria [384,385]. Mitochondrial SOD2 activity is reduced in preeclamptic placenta [309,386]. Mitochondrial ROS is also elevated in the kidney and placenta of RUPP rats [387]. Mitochondrial ROS in the placenta is raised by infusing/injecting with either of AT1-AA, sFlt-1, TNFα and ad-CMV-hypoxia-inducible factor (HIF) into pregnant rodents [302,388,389,390]. In cultured endothelial cells, serum from women with preeclampsia suppresses mitochondrial respiration and boosts mitochondrial ROS generation [391,392,393]. In a way like preeclamptic serum, sFlt-1 also promotes mitochondrial oxidative stress in endothelial cells [393]. Serum from RUPP rats similarly stimulates mitochondrial ROS production in HUVECs, which is ablated by AT1-AA inhibition [387,388]. Hypoxic treatment reduces mitochondrial respiration in cultured trophoblast-like JEG3 cells and increases mitochondrial ROS in human placenta [394,395]. In vitro hypoxia also suppresses mitochondrial respiration and increases mitochondrial ROS in ovine uterine artery smooth muscle cells [396].

### 4.3. A Causative Role of ROS in in Animal Models of Hypertension-Related Disorders

Oxidative stress is believed to be linked to pathogenesis and progression of a myriad of human diseases including hypertension-related disorders [21,23,24,397,398,399,400,401]. Numerous studies using animal models reveal a causative role of ROS in the pathogenesis of hypertension, diabetes, and preeclampsia.

Treatment with the SOD mimetic tempol reduces vascular ROS and lower blood pressure in animal models of essential and secondary hypertension [253,257,269,317,402,403,404], of diabetes [405,406,407,408], and of preeclampsia [293,294,346,409]. SOD1 deletion promotes development of hypertension, whereas the delivery of liposome-encapsulated SOD diminishes Ang II-induced hypertension [267,410].

NOX inhibitors apocynin and diphenyleneiodonium have been shown to decrease vascular ROS, improve vascular function, and lower blood pressure in animal models of hypertension-related disorders [272,275,312,317,324,340,411,412,413,414,415]. Angiotensin II-induced vascular ROS and hypertension is enhanced in transgenic mice overexpressing NOX1 in smooth muscle cells and is suppressed in NOX1 deficient mice [274,325,416]. Similarly, angiotensin II-induced vascular O_2_^•−^ and hypertension are blunted in p47^phox−/−^ mice [271]. Deletion of p47^phox^ prevents diabetes-induced vascular dysfunction in mice [332,417]. Similar findings are also observed in db/db mice with siRNA-induced p22^phox^ knockdown [334].

Administration of mitochondria-targeted antioxidants have extensively explored in animal models of hypertension and preeclampsia. MitoQ decreases blood pressure in SPSHR [418], whereas MitoEbselen reduces vascular ROS and prevents angiotensin II-induced hypertension in mice [360]. MitoQ and MitoTempol also reduce placental oxidative stress, lowers blood pressure, and improves fetal growth in RUPP rats and in pregnant rats experiencing gestational hypoxia [301,387,389]. Scavenging of mitochondrial isolevuglandins formed by peroxidation of arachidonic acid with Mito2HOBA and targeting cyclophilin D, a component of mitochondrial permeability transition pore, with its specific inhibitor sanglifehrin A reduce vascular mitochondrial ROS, improve vascular function, and attenuate hypertension in Ang II-infused mice [362,419]. These beneficial responses are replicated by overexpression of mitochondria-targeted SOD2 and catalase [362].

## 5. Contribution of Dysfunctional Ca_v_1.2 Conferred by ROS to Increased Vascular Tone in Hypertension-Related Disorders

### 5.1. ROS and Ca_v_1.2 Function/Expression

There is ample evidence that Ca_v_1.2 is regulated by cellular redox state. The α1c subunit contains 48 cysteines with some of them being sensitive to redox modulation [420]. Redox modulation of sulfhydryl groups of cysteine residues could alter the structure and function of proteins. Site-directed mutagenesis has identified several cysteine residues including C543, C1789, C1790, and C1810 that subject to redox regulation [421,422]. However, electrophysiological studies reveal conflicting effects of ROS on Ca_v_1.2. Studies from Amberg’s group demonstrate that bath application of H_2_O_2_ and ROS produced by xanthine oxidase/hypoxanthine stimulates Ca_v_1.2 activity in rat cerebral arterial smooth muscle cells [423,424]. Both mitochondria- and NOX-derived ROS could exert stimulatory effects on Ca_v_1.2. Antimycin enhances Ca_v_1.2 activity by promoting mitochondrial H_2_O_2_ generation. In addition, angiotensin II augments Ca_v_1.2 activity by promoting mitochondria- and NOX-mediated H_2_O_2_ production [423,424,425]. Rotenone also increases Ca_v_1.2-mediated currents by stimulating mitochondrial O_2_^•−^ production in A7r5 aortic smooth muscle cells [426]. In contrast, sulfhydryl-oxidizing agents 2,2′-dithiodipyridine (DTDP) and thimerosal inhibit heterologously expressed α1c subunit of rabbit smooth muscle Ca_v_1.2 [427]. In rat tail arterial smooth muscle cells, 2,5-di-t-butyl-1,4-benzohydroquinone inhibits Ca_v_1.2 via O_2_^•−^ generation [428]. H_2_O_2_ is found to inhibit Ca_v_1.2 in A7r5 aortic smooth muscle cells [426]. The disunity in electrophysiological findings suggests complexity of ROS actions, possibly due to concentrations of ROS used, the expression systems, ROS compartmentation and absence/presence of channel subunits/partners.

The redox modulation of Ca_v_1.2 has immense impacts on vascular function. H_2_O_2_ is reported to trigger an increase in [Ca^2+^]_i_ and vasoconstriction in rat mesenteric arteries, rat coronary arteries, rat aorta, and canine cerebral arteries [429,430,431,432,433,434,435]. H_2_O_2_-induced increase in [Ca^2+^]_i_ and contractile response are inhibited by the removal of extracellular Ca^2+^ and by Ca_v_1.2 inhibitors nisoldipine, nifedipine, diltiazem, and verapamil, suggesting that H_2_O_2_ activates Ca_v_1.2 in vascular smooth muscle cells [429,430,431,434]. Likewise, an ONOO^−^-induced increase in [Ca^2+^]_i_ is inhibited by nifedipine in mesenteric arteriolar smooth muscle cells [436]. Thrombin stimulates anincrease of [Ca^2+^]_i_ via activating Ca_v_1.2 in rat aortic smooth muscle cells, which is absent in NOX1 null cells [437]. As expected, vessels from SHR exhibits greater increase in [Ca^2+^]_i_ and vasoconstriction compared to WKY [431,432,433,435]. It should be noted that H_2_O_2_ also functions as an EDHF in other vascular beds including mouse and human mesenteric arteries, mouse aorta, and in porcine coronary microvessels and causes vasorelaxation [438,439,440,441]. Depolarization with high concentrations of KCl causes vasoconstriction by activating Ca_v_1.2 [442]. Paraquat and LY83583 enhance KCl-induced increase in [Ca^2+^]_i_ and vasoconstriction by increasing O_2_^•−^ production in rat renal afferent arterioles, mesenteric arteries and intrapulmonary arteries [443,444]. Sphingosylphosphorylcholine also potentiates the contractile response to KCl in a NOX-dependent mechanism in rat mesenteric and intrapulmonary arteries [443].

NO is also a reactive, gaseous signaling molecule. S-nitrosylation, the covalent attachment of NO to a cysteine thiol in a given protein, is also an important redox signaling [445]. S-nitrosylation of Cys 1180 and/or Cys1280 in Ca_v_1.2 reduces surface expression of the channel and channel activity [446]. In addition, S-nitrosylation of Ca_v_1.2 also reduces channel open probability [447]. Excess ROS leads to low NO bioavailability in hypertension [448]. Intriguingly, Ca_v_1.2 S-nitrosylation levels are reduced in aorta from SHR rats and in pulmonary arteries from patients with pulmonary hypertension [446]. Not surprisingly, increasing vascular s-nitrosylation levels of Ca_v_1.2 by injecting S-nitrosocysteine reduces blood pressure in SHR rats [446].

**Figure 4:**
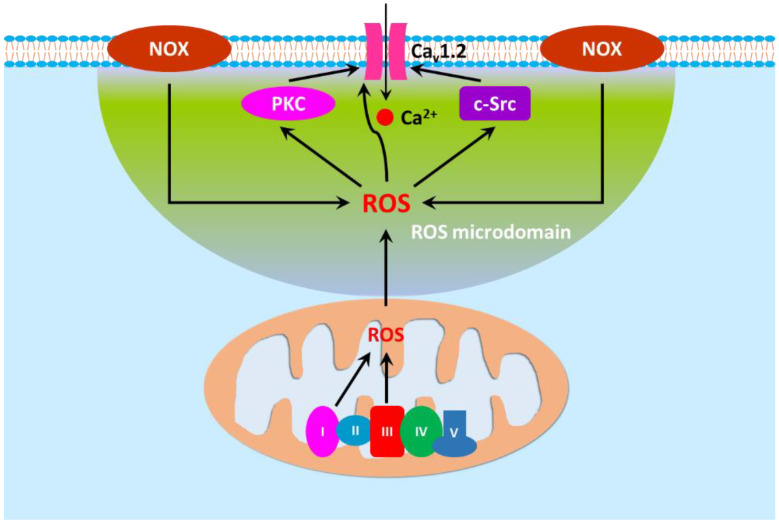
Regulation of Ca_v_1.2 by ROS microdomains. In vascular smooth muscle cells, ROS-derived from NOXs in plasma membrane and/or subcellular mitochondria in the immediate vicinity of Ca_v_1.2 channels form ROS microdomains. Ca_v_1.2 activity can be altered directly and indirectly by ROS. Within ROS microdomains, ROS can directly target cysteine residues in Ca_v_1.2 to modify channel activity. In addition, ROS can also trigger ROS-dependent activation of PKC and/or c-Src. Activated PKC and c-Src in turn phosphorylate Ca_v_1.2 and enhance channel activity.

ROS generated in different cellular compartments form microdomains and act on their targets in a spatiotemporal manner [449] (Figure 4). The redox regulation of Ca_v_1.2 requires adjacent location of mitochondria and NOXs to Ca_v_1.2 on the plasma membrane in vascular smooth muscle cells [423,425]. Thus, it is expected that microdomains with high concentrations of ROS exist in subcellular regions next to Ca_v_1.2. Interestingly, redox modulation of Ca_v_1.2 can occur directly or indirectly. In addition to directly targeting Ca_v_1.2, ROS could oxidize partners of Ca_v_1.2 to indirectly alter Ca_v_1.2 activity. As aforementioned, PKC and c-Src form a partnership with Ca_v_1.2 to regulate the activity of the channel. PKC contains six cysteine residues in each of the two pairs of zinc fingers within the regulatory domain and the oxidation of them activates PKCs [450,451]. H_2_O_2_ has been shown to activate PKCs in vascular smooth muscle cells [452,453]. SH2 domain of c-Src also contains cysteine residues and oxidation of these residues promotes the formation of disulfide bridges that subsequently override the autoinhibitory effect of Tyr527, leading to enzymatic activation [454]. Vascular c-Src is activated by NOX4- and mitochondria-derived ROS [287,455]. ROS produced by both NOXs and mitochondria are found to activate adjoining PKCs, which in turn enhances Ca_v_1.2 activity in vascular smooth muscle cells [423,425,426]. Not surprisingly, H_2_O_2_-induced vasocontraction is reduced by PKC/c-Src inhibition and by Ca_v_1.2 blockade [430,434,435,456,457]. Similarly, the potentiation of KCl-induced vasoconstriction of rat mesenteric and intrapulmonary arteries by NOX-derived ROS is also sensitive to the inhibition of PKC/c-Src [443]. Calmodulin is a key Ca^2+^ sensor for both Ca_v_1.2 inactivation and facilitation [458]. Modulation of calmodulin by H_2_O_2_ also augments cardiac Ca_v_1.2 activity [459].

Remarkably, ROS also regulate gene expression [460,461]. There is a concurring decrease in both vascular ROS and Ca_v_1.2 expression in aortas of aged mice lacking the mineralocorticoid receptor in smooth muscle cells [142]. The protein expression of the α1C subunit is increased by angiotensin II in cultured rat mesenteric arteries [462,463]. H_2_O_2_ mimics, while NOX inhibition by apocynin, diphenyleneiodonium and gp91ds-tat as well as catalase annuls angiotensin II-mediated upregulation of the α1C subunit [463]. In a cardiac muscle cell line HL-1 cells, angiotensin II upregulates the α1C subunit at both mRNA and protein levels through the NOX-PKC pathway-mediated cAMP response element binding protein (CREB) phosphorylation [464]. It is expected that this mechanism could also play a role in secondary hypertension as hypertension due to Cushing’s syndrome, hyperaldosteronism, and renovascular disease involves activation of the renin-angiotensin system [231,256,465,466,467,468,469]. In rat cerebral arteries, endothelin 1 promotes mitochondrial ROS generation which subsequently stimulates the expression of the α1C subunit by activating nuclear factor kappa B (NF-κB) [470]. Consequently, the upregulation of Ca_v_1.2 induced by endothelin 1 increases both myogenic tone and depolarization-induced vasoconstriction.

### 5.2. ROS and Myogenic Tone

Exogenous ROSs have been shown to alter myogenic tone. In pressurized mouse tail arterioles and rat gracilis skeletal muscle arterioles, H_2_O_2_ causes vasoconstriction [471,472]. The exposure of rat cerebral arteries to the ROS-generating system xanthine oxidase plus hypoxanthine also increases myogenic tone [423]. Likewise, O_2_^•−^ generated by paraquat enhances myogenic tone in mouse afferent arterioles [473]. ^•^OH generated by the Fenton reaction from H_2_O_2_ and the iron redox chelate Fe^3+^/nitrilotriacetate (FeNTA) elevates myogenic tone in denuded rat ophthalmic arteries [474].

Numerous studies reveal that endogenous ROS plays a key role in intralumenal pressure-induced myogenic tone. Myogenic tone in mesenteric arteries from SOD1 knockout mice is increased [475]. ROS derived from both NOXs and mitochondrial contribute to myogenic tone development. Pressurization of rat femoral arterial branches increases O_2_^•−^ via activating NOXs [476]. Similarly, an increase in intralumenal pressure promotes ROS in vascular smooth muscle cells and myogenic constriction simultaneously in mouse tail arterioles, which are inhibited by diphenyleneiodonium/catalase and are absent in arterioles from p47^phox−/−^ mice or transgenic mice expressing a dominant-negative mutant of human rac1 (rac-DN) [471]. Likewise, pressure-dependent myogenic tone enhanced in mouse afferent arterioles is repressed in vessels from p47^phox−/−^ mice and by NOX inhibitors apocynin and diphenyleneiodonium [477,478]. The regulation of myogenic tone by ROS is tissue-, ROS type- and ROS source-dependent. H_2_O_2_, but not O_2_^•−^, contributes to myogenic constrictor response in rat tail arterioles as the vasoconstriction is diminished by catalase, but not by manganese(III) tetrakis(1-methyl-4-pyridyl)porphyrin (MnTMPyP), a cell-permeant mimic of SOD [471]. However, myogenic tone is reduced by pegylated SOD, but not by pegylated catalase in mouse afferent arterioles [477]. Selective inhibition of both NOX1 and NOX2 with 2-acetylphenothiazine (2-APT) and VAS2870, respectively, suppressed basal myogenic tone in rat cremaster skeletal muscle arterioles [479], whereas myogenic tone is inhibited by NOX1 inhibitor ML171 but not by NOX2 inhibitor gp91-dstat in rat cerebral arteries [480]. The concomitant increases in superoxide/H_2_O_2_ and myogenic constriction induced by pressure is also reduced by MitoQ in rat cerebral arteries [481].

As expected, both O_2_^•−^ and myogenic tone are increased in vessels from animal models of hypertension-related disorders including SHR cerebral arteries/afferent arterioles, OZR gracilis skeletal muscle arterioles/cerebral arteries, and db/db mouse mesenteric arteries [131,138,482,483,484,485]. The increased myogenic constriction of mesenteric arteries of db/db mice is reduced by supplement of GSH or the Nrf2-activator sulforaphane [485]. A reduction in myogenic tone is also achieved by scavenging ROS in SHR afferent arterioles, db/db gracilis skeletal muscle arterioles, and OZR cerebral arteries/gracilis skeletal muscle arterioles [131,281,482,483,484]. Aging is associated with increased prevalence of hypertension and increases in intralumenal pressure promote mitochondrial oxidative stress in aged mouse cerebral arteries [486]. The exacerbated myogenic tone of uterine arteries from high-altitude pregnant sheep concurs with increased expression of NOX2 and mitochondrial ROS, and pressure-induced myogenic tone in uterine arteries from high-altitude pregnant sheep is diminished by apocynin/mitoQ, whereas it is enhanced in uterine arteries from low-altitude pregnant sheep by mitochondrial respiratory chain inhibitors rotenone and antimycin [299,396].

Numerous studies demonstrate that PKC/c-Src activation contributes to myogenic tone development in small arteries/arterioles [136,487,488,489,490,491]. The expression/activity of vascular PKC/c-Src is increased in SHR and STZ rats as well as in uterine arteries of high-altitude pregnant sheep [179,435,492,493]. PKC/c-Src activation has a greater contribution to myogenic tone in SHR cerebral arteries, STZ rat gracilis skeletal muscle arterioles, and high-altitude sheep uterine arteries [136,159,179]. Notably, the increased myogenic constriction by ROS in rat cerebral arteries is mediated by ROS-mediated PKC/c-Src activation [423,473,494]. Moreover, PKC/c-Src activation in turn stimulates Ca_v_1.2 activity to promote myogenic constriction [86,112,473]. Nevertheless, both PKCs and c-Src can also contribute to myogenic tone development by increasing the sensitization of the contractile apparatus to Ca^2+^ [112,136].

## 6. Conclusions/Perspective

Evidently, Ca^2+^ influx through Ca_v_1.2 is fundamental to vasoconstriction and myogenic tone of small arteries and arterioles. Antihypertensive drugs including Ca_v_1.2 blockers have been successfully used to the management of hypertension-related disorders [495,496,497,498]. Oxidative stress is a hallmark of hypertension-related disorders. However, it is still an open question whether oxidative stress is a cause or consequence of hypertension. The data reviewed here highlights critical roles of vascular oxidative stress in the pathophysiology of hypertension-related disorders. Vascular Ca_v_1.2 is targeted by excessive ROS directly and indirectly leading to exaggerated channel expression/activity. The dysfunction of Ca_v_1.2 ultimately results in increased myogenic tone and elevated blood pressure. In preclinical studies, ROS-induced myogenic tone is diminished by Ca_v_1.2 blocker nifedipine [473]. Moreover, apocynin is as effective as nifedipine in lowering blood pressure in an animal model of hypertension [411]. Regulation of Ca_v_1.2 by ROS primarily occurs in ROS microdomains [423,425]. In this context, selectively combating compartmentalized ROS could also be a viable therapeutic approach in the treatment of hypertension. Animal studies have provided compelling evidence supporting a causative role of ROS in the pathogenesis of hypertension-related disorders. However, clinical trials on humans provide less convincing evidence [23,499]. Although animal studies have significantly contributed to our understanding mechanisms underlying hypertension-related disorders, further work is needed to translate findings from animal research to clinical benefit.

## Data Availability

Data is contained within the article.
